# Consumer acceptance of a locally produced nutritive food bar in Lao PDR

**DOI:** 10.1186/s40795-026-01385-2

**Published:** 2026-07-03

**Authors:** Marco J Haenssgen, Eric Deharo, Nutcha Charoenboon, Philippe Schmidt, Toum Lathsamee, Jacques Berger, Frank T Wieringa

**Affiliations:** 1 Department of Social Sciences and Development, , Building 2, Faculty of Social Sciences, Chiang Mai University, 239 Huay Kaew Rd. T. Suthep, A. Mueang, Chiang Mai, 50200 Thailand; 2 UMR MIVEGEC, French National Research Institute for Sustainable Development (IRD), Montpellier, France; 3 Bristol Medical School, University of Bristol, Bristol, UK; 4 Mai Savanh Lao Co. Ltd, Vientiane, Laos; 5 Faculty of Pharmacy, University of Health Sciences, Vientiane, Laos; 6 UMR QualiSud, French National Research Institute for Sustainable Development (IRD), Montpellier, France; 7 QualiSud Université de Montpellier, CIRAD, IRD, Université dela Reunion, Avignon Université, Montpellier, SupAgro France

**Keywords:** Ready-to-use supplementary food, Nutrition, Acceptability study, Willingness to pay, Rural areas, Lao PDR

## Abstract

**Background:**

Food insecurity and micronutrient deficiency remain pressing global development challenges, particularly for women and children in poor and remote communities in low- and middle-income countries. Global interest has grown in locally produced ready-to-eat nutritive foods to help combat different forms of malnutrition. The aim of this paper was to understand the acceptability of a novel and locally produced nutritive food bar for rural consumers at risk of malnutrition in Lao PDR.

**Methods:**

This multidisciplinary cross-sectional acceptability study assessed the organoleptic qualities of five different nutritive food bar formulations; willingness to use ready-to-use foods; and nutrition knowledge, attitudes, and behaviours related to ready-to-use foods. The study, implemented in June 2023, used structured questionnaires administered by trained interviewers to elicit data from 62 subject pairs of children and their caregivers from four rural communities in Vientiane Province. The data were analysed using non-inferential descriptive statistical analysis (univariate and bivariate analysis).

**Results:**

All five bar formulations received organoleptic ratings from 3.7 to 4.1 (adults) and 3.7 to 4.0 (children) on a 1-to-5 scale (5 being ”very good”). 93.6% caregivers would buy their preferred bar in a shop and 91.9% would have their child buy it at school. Participants’ average willingness-to-pay for their preferred bar was LAK 2580.64 (US$ 0.14) per bar, which was less than the production cost (LAK 4300). 95.2% of caregivers had knowledge on micronutrients and 75.8% on food fortification. At least weekly consumption of ready-to-use foods in participants’ households was 93.5% for salty snacks, 95.2% for sweets, and 98.4% for drinks. Willingness-to-pay appeared to be linked to lower age, higher household-level ready-to-use food spending, social and mass media use for nutrition information, and previous experiences with nutritive food bars for children.

**Conclusions:**

We conclude that a locally produced nutritive food bar has potential to improve nutrition intake among vulnerable populations at risk of malnutrition, such as women in childbearing age or school-aged children. Since rural development may raise demand for convenient ready-to-use foods, a nutritive food bar could provide a healthier alternative for households with high existing consumption of salty snacks and sweets, aided by market-based instruments and social/mass media outreach.

**Supplementary Information:**

The online version contains supplementary material available at 10.1186/s40795-026-01385-2.

## Background

The global priority challenge of food and nutrition insecurity persists. According to 2024 data by FAO et al. [1], more than 685 million people worldwide remain undernourished, while 2.6 billion individuals globally cannot afford a healthy diet. Micronutrient deficiencies affect billions of people worldwide: Recent modelling estimates by Passarelli et al. [2] suggested that, in the absence of fortification and supplementation, between 5.1 and 3.6 billion people (68% to 51% of the world population in 2018) would have insufficient intake of at least one of several key micronutrients including iodine, vitamin E, Calcium, iron, riboflavin, folate, vitamin C, vitamin B6, and vitamin A. Stevens et al. [3] further highlighted distinct gender and age gradients, estimating that two out of three women and more than half of children under the age of five globally are deficient in one or more micronutrients.

The problem is particularly severe in low- and middle-income countries in Asia and sub-Saharan Africa, where the diets of vulnerable populations are often insufficiently diverse and deficient in both macro- and micro-nutrients [2, 4, 5]. The situation is further aggravated by a high prevalence of infectious diseases [6, 7], as well as by unsafe and contaminated food, the consumption of which has been responsible for 125,000 deaths among children below the age of five annually around the globe (reported by World Health Organization [8] in 2024 but based on 2010 data [9]) – concentrated in poor and remote settings with weak food, health, and transport infrastructural conditions. In short, widespread malnutrition, especially among women and children, continues to affect low- and middle-income countries in particular, and place considerable burden on their health systems, economies, and societies [10–12].

Nutritive ready-to-use therapeutic foods (RUTFs) and ready-to-use supplementary foods (RUSFs) are considered as a partial solution to these challenges (the latter having a lower energy density than the former; note that “supplementary” here in this article refers specifically to nutritive foods) [5, 13–16]. Linking high energy and nutrient density with food safety and the convenience of distribution, storage, and use, RUTFs and RUSFs have been effective in the fight against both acute and chronic malnutrition, and they have been advocated to support vulnerable populations at risk of malnutrition [15, 17–20]. Researchers around the world are therefore exploring various formulations of ingredients (for example, without dairy or with non-peanut lipid sources) and micronutrient fortification (for example, iron) to optimize the use of RUTFs and RUSFs [5, 15, 20–23].

Acceptability studies offer crucial insights into how both adult and child target populations perceive RUTFs and RUSFs – especially if produced locally as an alternative to imported products – and the underlying factors that contribute to variability in target group acceptance and willingness-to-pay [20, 21, 24–28]. This has important practical implications beyond the preparation of efficacy trials. It also allows for reflection on how to design interventions to optimise the subsequent scale-up of nutritional interventions, for instance, with the use of innovative behavioural design strategies [29–33]. Previous research has thereby demonstrated that (a) RUTFs and RUSFs can achieve high acceptability especially in settings where other forms of dietary supplementation lack consumer acceptance, that (b) children and caregivers may exhibit diverging preferences for taste profiles and RUTFs/RUSFs types, and that (c) production with local ingredients offers additional benefits for sustainability and acceptability [20, 24, 26]. Consumer acceptability studies are crucial to explore market opportunities for new food [34–36] – especially if the market introduction also follows a development objective [37–39].

This article contributes to the understanding of RUSFs in particular. We present the findings of a pilot study on the organoleptic qualities and acceptability of five nutritive food bars, based on local ingredients, which have been newly developed in Lao PDR. The primary objective of the study was to explore the appreciation of different formulations of a nutritive food bar among children and caregivers. Supplementary objectives were to gauge the willingness to use ready-to-use foods (RUFs) in general, and to assess nutrition-related knowledge, attitudes, and behaviours.

Note thereby that the nutritive food bars fall into the category of RUSFs, and both RUTFs and RUSFs fall into a broader category of ready-to-use foods (RUFs), which also include unhealthy and ultra-processed convenience and snack foods as well as sugar-sweetened beverages (SSBs) that have the potential to exacerbate the double burden of malnutrition in low- and middle-income countries. As this broader category of RUFs includes nutritive as well as unhealthy food categories, this article will explicitly refer to RUTFs/RUSFs and nutritive food bars when considering healthy RUFs, and ultra-processed/salty/sweet convenience/snack foods and SSBs when considering unhealthy RUFs. Nutritive RUSFs/RUTFs have the potential to play a role in combatting undernutrition in their own right, but also if they can take advantage of the growing interest in, and market demand for, RUFs in general and thus substitute unhealthy foods for nutritive alternatives. Appealing RUSF/RUTF qualities such as convenience, cost, taste profiles, and energy density can thereby meet a growing demand for RUFs while providing necessary nutritional value to children and other populations at risk of undernutrition. As the food bar studied in this research had a comparatively high sugar content of 9.5 g in each 30 g bar (see Methods section for ingredients and nutritional values) compared to other RUSFs/RUTFs, its testing was justified primarily by the substitution away from widespread unhealthy RUF consumption in Lao PDR and especially in school food environments, and by the potential to supplement the diets of rural populations at risk of undernutrition.

The rural study setting in Lao PDR is highly relevant for nutrition research on locally produced RUSFs such as the nutritive food bar studied in this research. According to latest available data from 2023 to 2024, more than one-third (35.6%) of the Lao population were classified as moderately or severely food insecure (compared to 5.4% in neighbouring Thailand) [1], one third of children under the age of five were stunted [40, 41], and 39.2% of women were anaemic [42]. Prior research has underlined that locally produced RUTFs and RUSFs can play potentially an important role for food and nutrition policy in Lao PDR [28]. Several factors contribute to this assessment: Firstly, locally produced foods have been argued to have higher acceptability than imported solutions in the Southeast Asian context [20, 24, 43]. Secondly, Lao PDR has recently been experiencing rapid rural development, which potentially aids the consumption of healthy market-based RUFs (and increasing the ratio of healthy vs. unhealthy RUFs). Thirdly, recent macroeconomic shocks including high inflation and an exchange rate slump have threatened to render non-local food products unaffordable for the most vulnerable segments of the population (official consumer price inflation reached 23.0% in 2022, 31.2% in 2023, and 23.1% in 2024) [44, 45]. The type of RUSF assessed in this study – a nutritive food bar – has also been considered to be of high potential acceptability in the Lao context: Comparing four RUTFs in the form of a nutritive food bar, compressed bean cake, paste, and a fish-paste-filled wafer among 83 caregiver-child pairs in two Lao provinces, Aiello et al. [28] found that the food bar format had the highest average acceptability and was most commonly (53.0%) rated the favourite product among the study participants. While ultra-processed foods, fortified food products such as fortified milk-based drinks, and imported nutritive food bars were already available in Lao PDR, rural residents were not commonly acquainted with nutritive food bars, and the specific food bar used in this study therefore represented a novel addition to the local food environment [46–48].

## Methods

### Research design

The present study was part of the NutriLao project, whose overall objective was to contribute to optimal nutrition by improving the quality, diversity, and sustainability of diets through the consumption of innovative, socially accepted, and locally produced nutritive food products. Here, we present the results of a cross-sectional study on organoleptic qualities and acceptability of these nutritive food bars within the broader context of RUFs. The study was conducted in June 2023 with children aged 2–16 years comprising the full spectrum of pre-school to high school ages, plus their caregivers. The research has been reviewed and approved by the Lao PDR University of Health Sciences Research Ethics Committee (ref. 460/REC 06/04/23).

### Study site

The research was implemented in Thoulakhom District in Vientiane Province, northern Lao PDR. Based on the latest available census data from 2015, the district had a poverty rate of 9.5%, a net primary school enrolment rate of 73.5%, and 92.4% of households had access to improved water sources [49]. Within Thoulakhom District, four rural communities in a 10 km radius and with a travel distance from Vientiane Capital city of approximately 60 to 90 min were selected. The four communities were ethnically homogenous (majority lowland Lao Loum population) but had varied levels of wealth, food insecurity, and remoteness to their nearest urban area.

The sites were selected based on existing, long-standing relationships with the study team from previous and parallel research projects [48], which enabled (a) sampling of adult caregivers living in households with at least one child aged between 2 and 16 years (see next section), (b) reinforcing relationships of trust from the communities as the acceptability study involved the introduction and testing of hitherto unknown food products, and (c) a better understanding of the study site context (for example, food consumption behaviour). As the site selection was non-random, the sites should be understood as purposively selected rural case study settings.

### Participants

Participants were recruited from across the four study communities using an existing sampling frame produced by the Activity Spaces and Household Exclusion from Food Environments project (project ref. IMMANA 3.03) [48]. This related project collected a random pro-poor sample in each of the four communities. This existing sample was drawn from a complete household register that was produced by each community’s village chief and contained information about the household registration, household wealth groups (low/medium/high), and ethnicity. The pro-poor sampling approach involved a simple random draw (with replacements) of 30 households in the ”low” and “medium” wealth groups, thus omitting “high” wealth households (for example, 25 out of 168 households in one of the study communities were such “high” wealth households). This pro-poor sampling approach in the related research was highly consistent with the development objective of this study to explore the acceptability of a nutritive food bar to support remote and vulnerable populations.

Based on available household member information (and considering that not all of the 30 households per community in the previous study included children), the present study selected a sub-sample with a target of at least 60 children (1 per household and at least 15 in each village) and their caregivers. While most acceptability trials have included children in the age range of 6 to 59 months, we opted for a broader age range of 2 to 16 years comprising the full spectrum of pre-school to high school ages as the tested product was trialled with view not only towards supplementing the diets of young children and mothers but also potentially replacing unhealthy snack foods with nutritive alternatives in Lao school food environments. 62 caregiver-children pairs were ultimately enrolled in the present study. The sample size corresponded to previous cross-sectional and short-term RUTF and RUSF acceptability studies [28, 50]. For example, studies reviewed by Marchini et al. [25] had a median sample size of 50 (with and without caregiver pairing). The inclusion and exclusion criteria for the participant recruitment were:

Inclusion criteria:

- Participants are from households participating in the Activity Spaces and Household Exclusion from Food Environments project.
- Children aged 2–16 years and their mothers or main caregivers.

Exclusion criteria:

- Any known specific food allergies or intolerance (for example, soy, nuts, or dairy allergy).
- Medical problems including, but not restricted to, severe acute malnutrition.
- Any medical condition that might interfere with obtaining a reliable assessment of taste preference.

### Food bars

The nutritive food bars used in the acceptability study were produced by the local social enterprise Maï Savanh Lao under the *Nyim Van* (“sweet smile”) brand specifically for the present study and were not commercially available. The food bars were made from local agroecological food resources with different proportions of ingredients including Sacha Inchi (*Plukenetia volubilis L.*, which has a high percentage of protein and essential fatty acids) [51–53], and contained fruit and peanuts, used to establish a variety of flavour profiles (banana and/or mango and/or hibiscus), mung bean flour, puffed rice, and glucose syrup (see Table 1 below). As the combination of ingredients resulted in nutritionally quite distinctly different food bars, each bar was fortified with a premix of vitamins and minerals providing 9 vitamins (vitamin A, B-vitamins, vitamin D) and 5 minerals (including Fe, Zn, and calcium) to ensure that they were all nutrient-dense. The ingredients were selected based on local and regional (cross-border) availability, their nutrient quality, and their organoleptic qualities (for example, hibiscus was added for its pleasant sour taste, whereas Sacha Inchi was added for nutrient quality through omega-3 fatty acids).

**Table 1:** Ingredients and nutritional value of the five food bars used in the acceptability study

Bar colour code	White	Green	Blue	Yellow	Purple
**Taste character**	Sour-fruity	Banana	Fruity	Nutty-banana	Nutty
**Ingredients**					
* Binder*	40%	40%	40%	40%	40%
* Mung bean*	10%	10%	4%	10%	10%
* Sacha Inchi*	2%	2%	4%	2%	2%
* Peanuts*	0%	10%	12%	14%	20%
* Popped rice*	15%	15%	15%	15%	15%
* Hibiscus*	10%	0%	6%	4%	0%
* Dried bananas*	22%	22%	16%	14%	12%
* Dried mango*	0%	0%	2%	0%	0%
* Vitamin premix*	1%	1%	1%	1%	1%
**Nutritional value**					
* Kcal/100g*	392	450	435	424	455
* Protein (%)*	6.1%	12.4%	9.5%	11.2%	13.2%
* Lipids (%)*	8.3%	18.5%	15.7%	14.7%	19.3%

All values are on “wet matter basis,” that is, the value obtained from the prepared, ready-to-eat, bar

**Fig. 1:**
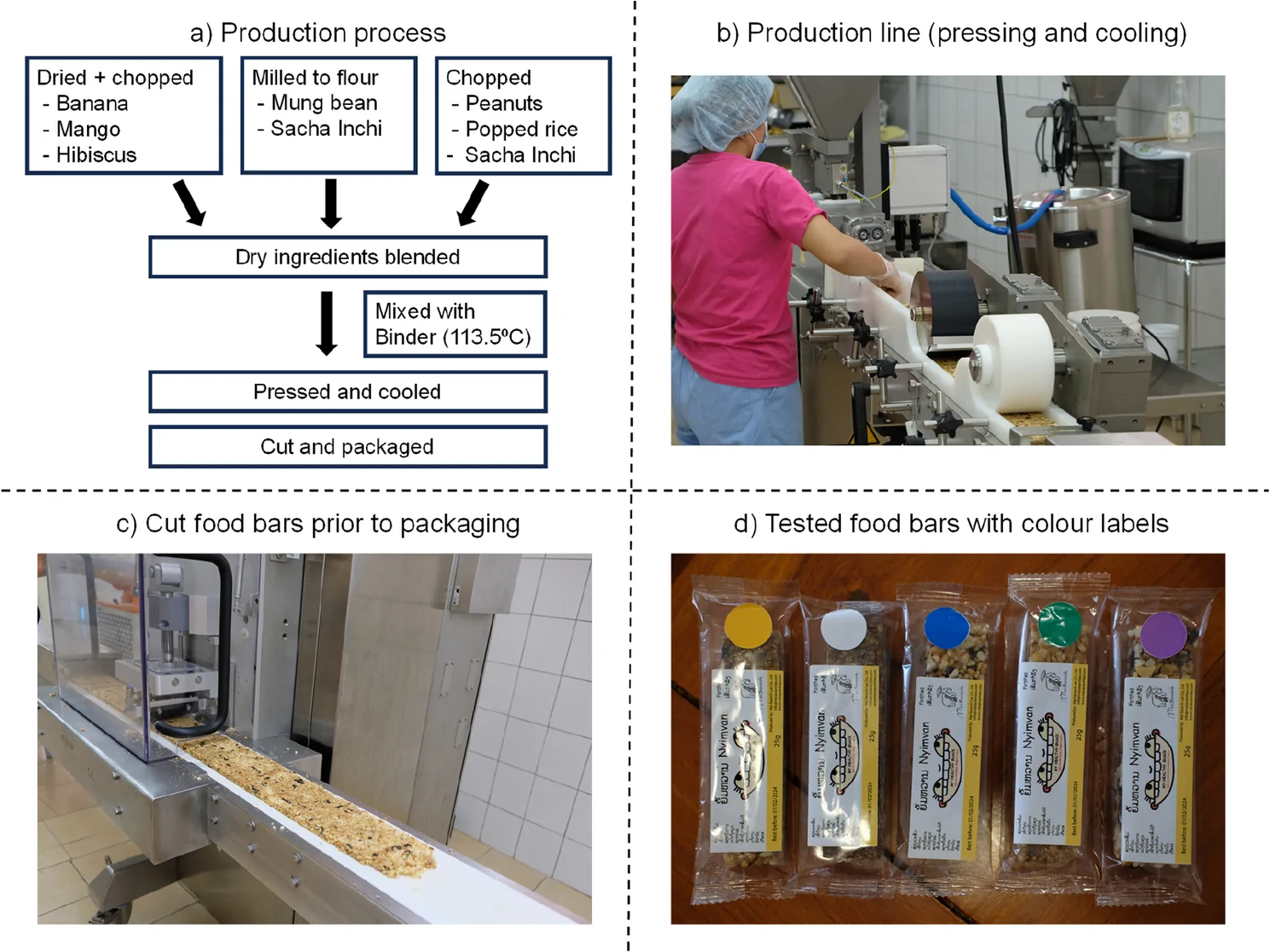
Production of the trialled food bars. Source: Authors

The overall production process of the food bars is illustrated Fig. 1, which presents the production process (Panel a) as well as the production line showing the process steps of pressing and cooling (Panel b) and cutting (Panel c). The food safety of the bar was certified by independent microbiological analysis (Eurofins document no. EVN-P-AR-FO3559), and Maï Savanh Lao further maintained quality certifications for making food products for human consumption (Organic Agriculture Certification Thailand no. 64-226-R1) and for its agri-food management system (Bureau Veritas Certification no. TH018885). The average production cost was LAK 4300 or approx. US$ 0.20 per bar.

The study compared five different recipes, and each bar type was marked with a colour label sticker (Table 1; shown in Fig. 1, Panel d with the colour labels). While the bars were similar in appearance, their taste varied across nutty, banana, and fruity character depending on the amounts of peanuts, banana, mango, and hibiscus. In terms of nutritional value (on “wet matter” basis obtained from the prepared bar), the energy content of the five bars ranged from 392 to 455 kcal/100 g, their protein content from 6.1% to 13.2%, and their lipid content from 8.3% to 19.3%. The total carbohydrate content of the bars was 64.0%, whereby added sugar was contained in the glucose syrup binder and accounted for 31.6% of the bars (3.7% saccharose and 27.9% glucose), or approximately 9.5 g in each 30 g bar.

### Data collection

Data were collected through a face-to-face survey using a structured digital questionnaire that took 40 min to administer (including an acceptability experiment, enclosed in the Supplemental Material), which was administered on tablet computers running the Kobo Toolbox software.

Following a two-day survey training, teams of four community-based enumerators (experienced in nutrition-related survey data collection in the same communities) implemented the study under the supervision of two Lao survey supervisors who had implemented survey research in the same study communities previously. Prior to the study implementation, the study team met district- and village-level authorities to inform them of the study and requested permission to conduct research activities in the rural communities. All caregivers in the sampled communities were subsequently invited to an introductory meeting where the study was explained and where they could ask questions (attended by a medical doctor to explain the nutritive character of the food bar and to build trust about participation in the study). During the informed consent procedure prior to enrolment, caregivers were informed again about the purpose of the study and asked for their consent to participate in the study both for the child under their supervision and themselves. The participants received a small gift as a token of appreciation for their participation.

The questionnaire was translated into Lao but administered by the enumerator, as literacy was varied in the local community. However, the ethnic and linguistic homogeneity of the study communities minimized language difficulties and further meant that variation in cultural factors would not impact sensory perception.

The acceptability study had two main components: direct acceptability testing of the five food bars among caregivers and children, and a complementary survey component involving only caregivers (not children). As the survey was conducted at the participants’ homes, the environment was not specially controlled but represented a real-life setting in which participants could comfortably test the bars and consider their responses. Acceptability testing included blinded testing (no prior knowledge of the ingredients) of the five food bars among children and their caregivers. To reduce potential biases in the assessment, the bar order was randomised individually for both children and caregivers using the in-built randomisation algorithm of the Kobo Toolbox software (see Fig. S1 in the Supplementary Materials for diagnostic data on the resulting bar sort order by colour and participant group). The organoleptic qualities were assessed across the dimensions of colour, smell, taste, appearance, chewiness, and hardness plus an overall impression. Adhering to common practice in RUF acceptability studies and to facilitate the data collection with children [25], the assessment used a five-point pictorial hedonic scale illustrated with smiley face representations from [1 – very bad] to [5 – very good].

Each participant received a sealed set of bars, which meant that ten bars were administered per child-caregiver pair. The bars were retrieved from a cooler to maintain quality. Every test involved a bite-sized portion that was broken off from the bar (approx. 25% or 7–8 g). After each tasting, the participants were asked to assess the organoleptic qualities of each bar and to clean their palate with a small cup of water. Feedback on the organoleptic qualities was provided by caregivers and children, who indicated the corresponding smiley face expression along the various organoleptic qualities. The interviewer provided instructions for interpretation of the 5-point scale and smiley face representations (which, pre-test and survey observations showed, were readily understood by caregivers), and would also explain it together with the caregiver to the child. The interviewer subsequently ensured that children indicated their responses independently without the caregiver’s influence.

The rank test was conducted for each individual after they completed the organoleptic quality assessment, selecting the bar that they liked the most and the least. While the use of pictorial smiley faces generally aids inclusiveness of children and low-literacy groups in acceptability studies [25], the rank test provided additional triangulation of bar acceptability in the case that diverse respondents (for example, young children, older children, adults) interpreted the gradients in the 5-point hedonic scale systematically differently.

The additional survey component involving only caregivers included the socio-demographic background of the respondent (gender, age, education), use of RUFs in general (with emphasis on unhealthy RUFs, considering frequency, household spending, and the rationale for consuming various RUFs such as salty snacks), and nutrition-related knowledge, attitudes, and behaviours (incl. prior experience with healthy RUFs). We also elicited more detailed information on the respondent’s preferred food bar: To reduce complexity, we opted for direct elicitation of the respondent’s willingness-to-pay for the preferred food bar (amount in Lao kip) and their willingness to (hypothetically) buy the preferred bar in a local village shop or letting their child buy it at school. The short module on questions about nutrition knowledge, attitudes, and practices focused specifically on micronutrients and food fortification (with an emphasis on knowledge and information sources).

The data were analysed descriptively in Stata 16. This included summarizing individual variables and exploring observed patterns between variables. No inferential statistical testing was conducted, consistent with the purposive (non-probabilistic) case study design. To support the interpretation of the acceptability test results, we also we assessed correlations between caregiver and children’s ratings to test for independence, and we explored if the randomisation order of the bars or the consumption of sweets were linked to the selection of the most preferred bar formulation. The latter indicatively used Pearson Χ^2^ tests for comparison of individual bar order [green, blue, and so forth] as ordinal variable and preferred bar choice as categorical variable, but without implying claims about the broader inference to the study population. These robustness checks were carried out separately for adults and children.

## Results

### Socio-demographic characteristics and nutrition-related knowledge, attitudes, and practices

The data gathered from the taste experiment involved 62 caregiver-child pairs from all four study communities (124 individuals in total), whose background characteristics are summarised in Table 2. All caregivers were the mothers of the children, with an average age of 46.7 years. A relative majority of the caregivers (45.2%) had a lower-secondary level of education, while 38.7% had primary-level education or below (this latter measure is the binary education indicator that we will use for simplicity in the subsequent analysis). Among their children, 59.7% were female with an average age of 8.3 years (ranging from 3 to 16 years). Almost all children (96.8%) were enrolled in school or pre-school education at the time of the data collection.

**Table 2:** Description of acceptability study sample

Variable	Caregiver	Child
***N*** **(% female)**	62 (100.0%)	62 (59.7%)
**Age (years ± SD)**	46.71 ± 12.72	8.27 ± 3.41
**Education**		
* No formal education*	9.7%	
* Primary / elementary*	29.0%	
* Lower secondary*	45.2%	
* Upper secondary*	11.1%	
* Tertiary / university*	4.8%	
* Currently in school (1 = yes)*		96.8%

The participants were familiar with food fortification: 95.1% had heard of minerals and vitamins and considered them “important.” 79.0% reported that they had ever taken food supplements, while 75.8% had heard about the specific term “food fortification.” To gather nutritional information, the respondents listed TV and radio (69.4%) and social media (62.9%) as main sources, followed by family members (46.8%) and private as well as public healthcare providers (private clinics: 41.9%, public hospitals: 38.7%, primary healthcare centres: 37.1%). At the same time, 98.4%, of participants had given their children milk fortified with vitamins and minerals, and 62.9% had also provided them with micronutrient powder such as Superkid. 9.7% of the participants also reported having given micronutrient-fortified food bars to their children previously.

In terms of consumption behaviour of unhealthy RUFs, the average weekly household spending on any RUFs (but primarily including unhealthy RUFs such as snack foods and SSBs) was LAK 265,322.60 (US$ 14.47; exchange rate of 18,340 LAK/USD at mid-point of data collection on 21 June 2023; www.oanda.com). For comparison, the minimum wage for a person during the same period was equivalent to LAK 325,000 (US$ 17.72, or LAK 1,300,000 per month) [54]. Table 3 underlines this point: Nearly two-thirds of the households consumed salty snacks (for example, prawn crackers, potato chips, spicy seafood snacks) and sweets (for example, sweet Lao desserts, chocolates, jelly) daily, and 45.2% of the participants’ households consumed SSBs daily (in the local markets, usually sugary soft drinks). Less common was the consumption of instant noodles (as noodles could be purchased locally at food stalls and in restaurants) and the more “Western” (and thus more expensive) RUF type of cookies and cake. Convenience (85.5%) and availability (79.0%) were the most frequently cited reasons for consuming RUF and drinks, whereas price was least commonly cited (33.9%, see Table 4).

**Table 3:** Household consumption of ready-to-use foods and drinks

Frequency of consumption	Salty snacks	Sweets	SSBs	Instant noodles (prepared at home)	Cookies and cake
**Daily or more often**	66.1%	64.5%	45.2%	16.1%	0.0%
**A few times per week**	27.4%	30.6%	53.2%	75.8%	12.9%
**A few times per month**	6.5%	3.2%	1.6%	8.1%	37.1%
**Less than monthly**	0.0%	1.6%	0.0%	0.0%	45.2%
**Never/rarely**	0.0%	0.0%	0.0%	0.0%	4.8%
***n***	62	62	62	62	62

Consumption by any household member, regardless of whether people eat/drink these foods individually or together

**Table 4:** Cited reasons for or against consumption of ready-to-use foods and drinks

Reason	Reason *for* RUF consumption	Neutral / no opinion	Reason *against* RUF consumption
Convenience	85.5%	14.5%	0.0%
Quench thirst/hunger	64.5%	35.5%	0.0%
Availability	79.0%	21.0%	0.0%
Taste	62.9%	37.1%	0.0%
Price	33.9%	64.5%	1.6%
Healthiness	64.5%	33.9%	1.6%
Spending time with friends	62.9%	33.9%	3.2%

### Ranking outcomes

**Fig. 2:**
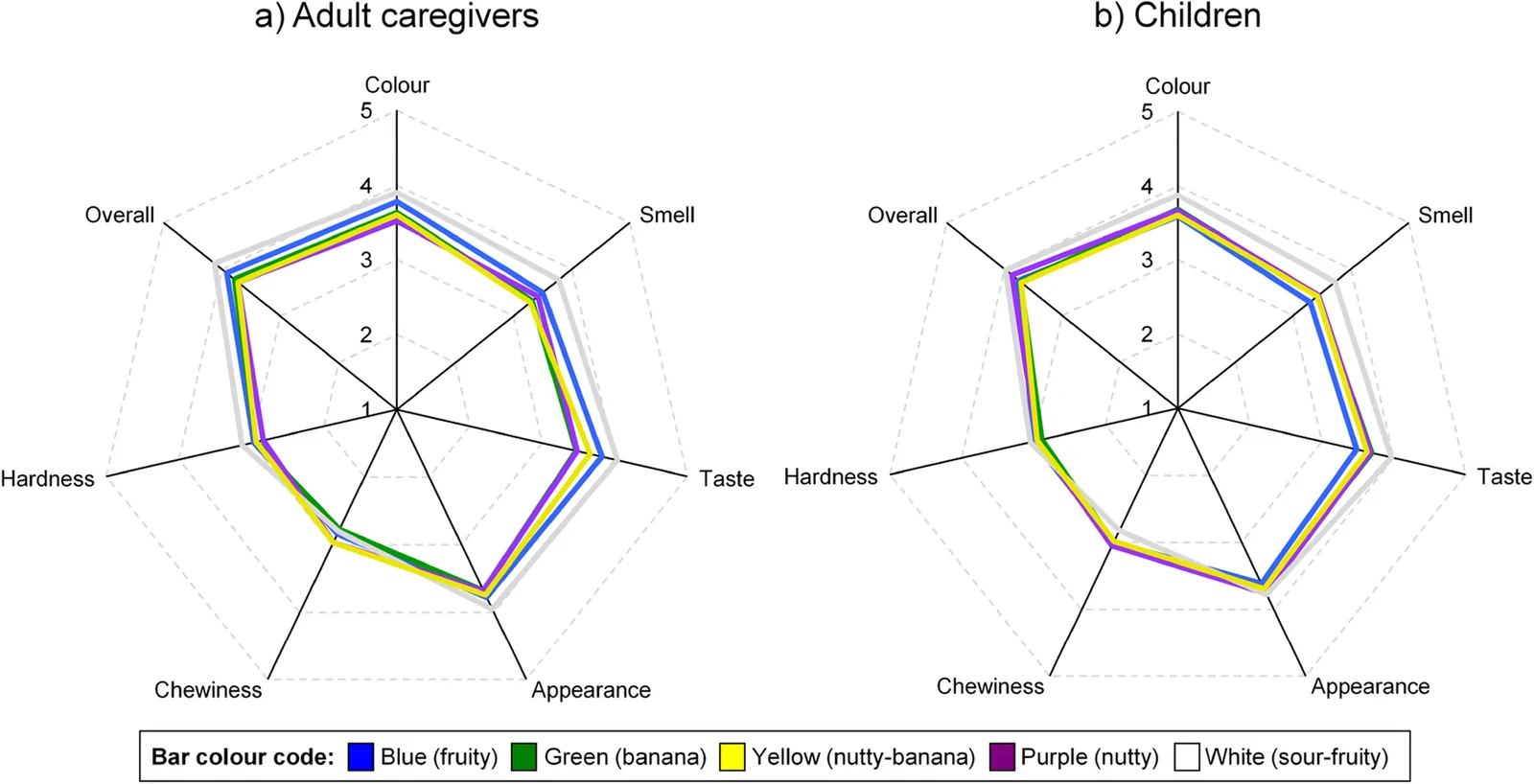
Radar plots of organoleptic rating among adult caregivers and children. *Notes.* *N* = 124 (62 adults, 62 children). Colours corresponding to food bar codes. Organoleptic qualities rated on a five-point hedonic scale from [1 – very bad] to [5 – very good]

Outcomes of the organoleptic quality rating are shown in Fig. 2. On a scale from [1 – very bad] to [5 – very good], adult caregivers provided an average overall rating from 3.7 to 4.1 across the five bars, while children rated them from 3.7 to 4.0. The white bar received the highest rating in both groups, and the yellow bar the lowest (tied with purple in the adult group). The different components of the organoleptic qualities (colour, smell, taste, appearance, chewiness, and hardness) exhibited only marginal differences between the five bars, whereby the white bar again tended to receive the highest average scores across the various categories. Across the categories, the participants tended to score colour, taste, and appearance above the mid-point of the scale at 3.0 (3.7 on average across all adult and child respondents), whereas the average rating for hardness fell onto the mid-point at 3.0, and chewiness was rated at 2.9 on average.

**Fig. 3:**
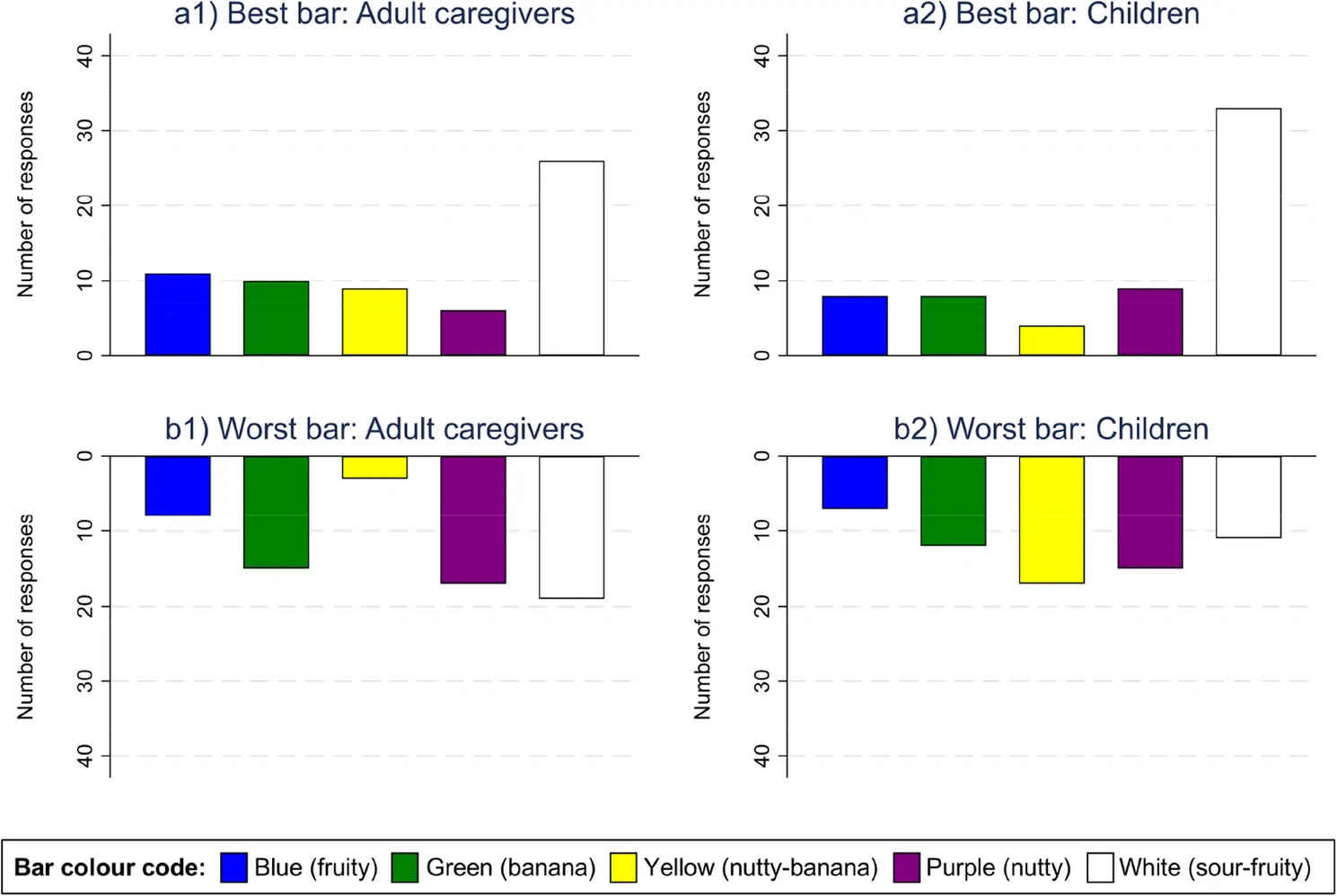
Overall ranking of bars. *Notes.* *N* = 124 (62 adults, 62 children). Colours corresponding to food bar codes

The rank test (selecting the “best” and “worst” among the five tested bars) showed that both groups selected the white bar with its sour-fruity taste character as the most liked one. This was the case for 41.9% of adult caregivers and 53.2% of children, whereby younger children more commonly selected the white bar than older children (68.8% of 16 children aged between 3 and 5 years, 50.0% of 24 children between 6 and 9 years, and 45.5% of 22 children between 10 and 16 years). Surprisingly, the white bar was also most often mentioned as the least liked bar among the adult caregivers (30.7%), while the least liked bar among children was the yellow bar (27.4%, nutty-banana taste character). Unlike adults, children did not commonly dislike the white bar (17.7%). The purple bar (nutty taste character) was the second-most frequently disliked bar among adults (27.4%) and children (24.2%), and thus also the most disliked bar overall. The least polarising option was the blue bar (fruity taste character), which ranked as the second-most liked bar overall and was relatively rarely disliked. The results of the rank test are summarised in Fig. 3.

Diagnostic data from the taste trial (see Fig. S1 in the Supplementary Materials) indicated that the randomisation sort order of the bars did not influence the rating outcomes: For example, the white bar appeared only between seven and ten times as either first or last among the five bars presented to adults and children, but it was by a large margin the preferred bar and also the least liked bar among adult caregivers. Indicative analysis between bar order and ranking outcomes showed no statistically significant association (using Pearson Χ^2^ tests for comparison of individual bar order [green, blue, and so forth] as ordinal variable and preferred bar choice as categorical variable). This also suggests that the sour-fruity white bar following sweeter bars did not evidently influence participants’ taste preferences. In addition, overall bar ratings between caregivers and children were not or only weakly correlated, with correlation coefficients ranging from − 0.04 [white bar] to + 0.26 [purple bar], indicating that children gave their responses independently without the caregivers’ influence.

### Acceptability

Acceptability indicators showed that more than 90% of the adult caregivers indicated that they would buy their preferred a bar in a shop (93.6%) or have their child buy it at school (91.9%). The average willingness-to-pay for the respondents’ preferred bar was LAK 2580.64, equivalent to US$ 0.14, and ranged from LAK 1000 to 5000 with most responses being LAK 2000 (46.8%) and LAK 3000 (33.9%). Recall for reference that the production costs of the bar were LAK 4300, while a pack of the popular chocolate wafer *beng-beng* (which has a similar size and to which respondents would occasionally compare the bar) would typically cost LAK 4000–5000.

### Covariates of acceptability

In the final step of the analysis, we described the background characteristics of the participants according to their preferred bar and examined the adults’ willingness-to-pay for their preferred food bars. Table 5 presents for each preferred bar choice the reported willingness-to-pay and characteristics of the respondents. Age differences among the respondents preferring the various bars were relatively minimal (with the yellow bar being a mild outlier as it was preferred by relatively younger adults and children). Among children, the blue bar was most preferred by girls (87.5%), while the children who liked the yellow bar had a 50–50 sex composition. Among adults, the yellow bar was liked by respondents with lower education (66.7% with primary education or below), whereas the green bar was preferred by respondents with higher education levels on average. Furthermore, adult participants preferring the yellow bar had the highest average willingness-to-pay (LAK 2,777.78, US$ 0.15) whereas participants preferring the blue bar had the lowest willingness-to-pay (LAK 2272.73, US$ 0.12).

**Table 5:** Willingness-to-pay and socioeconomic attributes by preferred bar

Preferred food bar	Caregiver					Child		
	Willing-ness-to-pay (LAK)	Age	Education (% primary or below)	Weekly household spending on RUF/drinks (LAK)	*N*	Age	Sex (% female)	*N*
**Blue (Fruity)**	2272.73	46.9	45.5%	230,000	11	8.4	87.5%	8
**Green (Nutty-banana)**	2400.00	48.4	20.0%	368,000	10	8.8	75.0%	8
**Yellow (Banana)**	2777.78	42.9	66.7%	255,556	9	6.8	50.0%	4
**Purple (Nutty)**	2666.67	46.8	50.0%	220,000	6	9.9	55.6%	9
**White (Sour-fruity)**	2692.31	47.3	30.8%	254,615	26	7.9	51.5%	33
**Total**	2580.65	46.7	38.7%	265,323	62	8.3	59.7%	62

The willingness-to-pay among the adult caregivers (Table 6) further appeared to be correlated with their socio-demographic background characteristics. Adult respondents with the lowest range of willingness-to-pay (LAK 1000–2000) had above-average education levels and below-average household expenditure on RUFs. In contrast, the group with the highest willingness-to-pay (LAK 4000–5000) had the lowest average age and the highest household expenditure on RUFs. Household consumption of sweets, cakes, or salty snacks was not systematically linked to adults’ or children’s choice of the preferred food bar or to adults’ willingness-to-pay for their preferred bar. It further appeared that willingness-to-pay had a positive relationship with the use of social media and TV/radio as sources for nutrition information, and with the experience of giving their child micronutrient bars previously.

**Table 6:** Mean characteristics of adult caregivers by their willingness to pay for their preferred bar

	Willingness-to-pay (LAK)		
	1000–2000	3000	4000–5000
**Age**	48.3	48.7	33.3
**Education (% primary or below)**	35.3%	42.9%	42.9%
**Weekly household spending on RUF/drinks (LAK)**	227,353	282,857	397,143
**Household consumption of unhealthy RUFs (% yes)**			
* Carbonated drinks: Few times per week*	52.9%	52.4%	57.1%
* Daily*	47.1%	42.9%	42.9%
* Instant noodles: Few times per week*	79.4%	76.2%	57.1%
* Daily*	17.6%	9.5%	28.6%
* Sweets: Few times per week*	35.3%	28.6%	14.3%
* Daily*	64.7%	61.9%	71.4%
* Cookies and cake: Few times per week*	17.6%	4.8%	14.3%
* Daily*	0.0%	0.0%	0.0%
* Salty snacks: Few times per week*	26.5%	28.6%	28.6%
* Daily*	73.5%	61.9%	42.9%
**Sources of nutrition information (main sources; % yes)**	52.9%	52.4%	57.1%
* Family members*	50.0%	47.6%	28.6%
* TV / radio*	64.7%	71.4%	85.7%
* Social media*	55.9%	61.9%	100.0%
* Private clinic*	47.1%	38.1%	28.6%
* Health centre*	47.1%	28.6%	14.3%
* Public hospital*	44.1%	28.6%	42.9%
**Selected micronutrient practices**			
* Has personally taken supplements (% yes)*	82.4%	76.2%	71.4%
* Child received micronutrient powder (% yes)*	58.8%	71.4%	57.1%
* Child received micronutrient bars (% yes)*	5.9%	9.5%	28.6%
***n***	34	21	7

## Discussion

Drawing on a pro-poor sample of 124 caregivers and children across four rural communities in Vientiane Province, the study demonstrated a consistently moderate-to-high level of appreciation for all five food bar formulations, whereby assessments of appearance, taste, colour, and smell were favourable alongside moderate assessments of hardness and chewiness. The most liked bar formulation with a sour-fruity taste character (no peanuts and relatively high hibiscus and banana content; white colour-code) was also the most polarising among the participants, whereas the second-most liked formulation with a fruity taste character (less banana, hibiscus, and mung bean but relatively more peanuts and dried mango; blue colour-code) was also the least commonly disliked formulation. As the children’s hardness assessments of the bars covered the full spectrum from “very bad” to “very good,” it is also important to consider limiting the target group of the bar to children aged at least three years to prevent choking hazards [55].

Caregivers’ willingness to buy their preferred bar formulation either for themselves or for their children exceeded 90%, with an average willingness-to-pay of LAK 2580.64 (US$ 0.14) – both of which was similar to other RUTFs and RUSFs trialled in the region [24]. The analysis of nutrition-related knowledge, attitudes, and behaviours further demonstrated that caregivers were often familiar with micronutrients and food fortification, and the majority had previously provided micronutrient-fortified milk and powders to their children. At the same time, household-level RUF consumption, especially of sweets and salty snacks but also SSBs, was high. Total household spending on RUFs and drinks nearly reached the level of a worker’s minimum wage. Willingness-to-pay for the preferred bars varied across recipes and appeared to be linked to lower age, higher household-level RUF spending, the use of social and mass media for nutrition information, and previous experiences with food bars as RUSF for children.

The patterns suggest that younger respondents may prefer convenience foods because of lifestyle reasons and less available time due to work, which could drive their willingness-to-pay. Age and household expenditure on RUFs showed indeed a clear relationship with willingness-to-pay (correlation coefficients of − 0.28 and + 0.31, respectively), and the group with the highest willingness-to-pay had 74.7% higher weekly RUF spending than the lowest willingness-to-pay group. However, the data remained inconclusive on this interpretation owing to the lack of individual-level spending and consumption data. This was evident in that expenditure (as a household-level variable) was not or only weakly correlated with the individual-level variables of education and age (correlation coefficients of − 0.16 and − 0.04, respectively).

Although all bars were rated moderately highly and had high levels of acceptance, the scores in our organoleptic quality assessment appeared to be lower than those for non-local RUTFs reported by Aiello et al. [28] in Lao PDR (but higher than those reported in Vietnam [50]), which may partly be a result of the somewhat unfamiliar format of the food bar in the local food environment, different study locations within Lao PDR, or different taste profiles. At the same time, our study highlighted the relevance and synergies of introducing a locally produced nutritive food bar in the rural Lao context as an RUSF: such a product introduced into local markets has the potential to substitute at least partially for unhealthy salty and sweet snacks that dominate the high levels of household RUF spending in a challenging macroeconomic environment. This potential was further reinforced as the willingness-to-pay for such bars was also highest among the households with the highest weekly spending on RUFs and drinks. It further appeared that exposure to modern media could be a promising pathway to raising households’ willingness-to-pay for the nutritive food bar, for instance, through health information campaigns or marketing outreach by social enterprises producing RUSFs and RUTFs locally. However, health communication and market-based advertising activities need to be conscious of the risk that promoting the nutritive food bar as a healthy RUF could raise the willingness-to-pay and -to-buy not only for the food bar itself, but also for convenient processed foods more broadly – including unhealthy RUFs.

In practical terms, the study helped identify promising formulations to maximise the acceptability of the nutritive food bar. Specifically, while caregivers and children appreciated the organoleptic qualities of all bars, the taste experiment allowed us to detect more and less polarising formulations: while the sour-fruity (white) bar formulation was by far the most liked, it also had the potential to discourage use by a substantial portion of the target group. The fruity (blue) as the least commonly disliked bar represented a larger common taste denominator, and was therefore adopted in a subsequent efficacy trial that followed this pilot study in December 2023. However, the practical implications of this choice also relate to willingness-to-pay, which was 15.6% lower than the most-liked sour-fruity (white) bar, meaning that subsequent scale-up efforts would have to pay particular attention to either raising target groups’ willingness-to-pay (considering the aforementioned caveat of inadvertently raising willingness-to-pay for other ultra-processed and unhealthy RUFs) or to ensuring sufficiently efficient production and pricing / cross-subsidy models to meet the given willingness-to-pay expectations.

The limitations of this study point to opportunities for further research. Firstly, diagnostic data from the taste trial indicated that the randomisation sort order of the bars did not influence the rating outcomes. Although this suggests that the results are robust, our ability to capture correlates of acceptability and willingness-to-pay as well as the external validity and replicability of the study results with only four purposively sampled case study sites and a small sample of 62 caregiver-child pairs remained limited (also in light of recall biases as caregivers were for example commenting on common household-level consumption patterns among several household members). While our findings can be compared to other research on RUSFs and RUTFs in Lao PDR [28], future acceptability assessment should cover the diverse multi-ethnic settings and diverse food environments across Lao PDR, ideally in relation to broader RUF consumption in real-life purchasing and consumption situations. Furthermore, the assessment of organoleptic quality and willingness-to-pay could be strengthened by including a control food bar with known acceptability for comparative purposes. Longer-term observational data collection is further advisable to gauge the persistence of acceptability over time [28, 50], for instance, as the novelty of a locally produced food bar may gradually wear off and as communities’ experience with food aid and food fortification programmes may lead them to overstate their acceptability in face-to-face surveys. Direct elicitation of willingness-to-pay also has a hypothetical bias that can be overcome in real-choice experiments involving competing (healthy and unhealthy) RUFs and variations of presentation and packaging [56], the latter of which can be optimised through cognitive research that utilises, for instance, laboratory-based eye-tracking studies [57, 58]. In addition, the relatively high added sugar content of the bars is a limitation – affecting both the nutritive character and potentially influencing the acceptability of the bar – but should be considered in light of the bars’ role as an RUSF. In an everyday market-based environment, the bars have the potential to substitute consumption away from the unhealthy salty snacks and sweets that dominate the daily food patterns in the study area, and can thereby render nutritional profiles more favourable.

## Conclusions

Our study contributes to the growing research and policy interest in locally produced RUSFs as a tool to improve the intake of nutrients for prevention of malnutrition and support at-risk population groups [20, 21, 24–27]. Using a nutritive food bar as an example of such RUSFs in Lao PDR, the study offers important programme-relevant information about local taste preferences, sociodemographic covariates of acceptability, and synergistic options to introduce the nutritive food bar in a local food environment – as it was dominated by high consumption RUFs already, although mainly comprising salty snacks and sweets. In the present case, the study results led the research team to adopt a non-polarising fruity bar formulation for further efficacy testing to optimise the acceptability of the product among the largest possible target group. As a product created in cooperation with a local social enterprise, the food bar also has the potential for market-based strategies that support vulnerable groups in the general population with risk of malnutrition, such as women of childbearing age or school-aged children. Our study further documented differences in preferences for various bar recipes among female caregivers and children. These heterogeneities also offer opportunities to target vulnerable population segments with group-specific product characteristics (for example, recipes and packaging) to stimulate the uptake of market-based RUSFs yet further.

## Supplementary Information

Supplementary Material 1.

## Data Availability

The data presented in this study are available on request from the corresponding author.

## References

[1] FAO, IFAD, UNICEF, WFP, and WHO. The state of food security and nutrition in the world 2025: addressing high food price inflation for food security and nutrition. FAO; IFAD; UNICEF; WFP; WHO: Rome; 2025.

[2] Passarelli S, Free CM, Shepon A, Beal T, Batis C, Golden CD. Global estimation of dietary micronutrient inadequacies: a modelling analysis. Lancet Global Health. 2024;12(10):e1590–9. 10.1016/s2214-109x(24)00276-6. 39218000 10.1016/S2214-109X(24)00276-6 PMC11426101

[3] Stevens GA, Beal T, Mbuya MNN, Luo H, Neufeld LM, Addo OY, et al. Micronutrient deficiencies among preschool-aged children and women of reproductive age worldwide: a pooled analysis of individual-level data from population-representative surveys. Lancet Global Health. 2022;10(11):e1590–9. 10.1016/s2214-109x(22)00367-9. 36240826 10.1016/S2214-109X(22)00367-9 PMC10918648

[4] Bechoff A, de Bruyn J, Alpha A, Wieringa F, Greffeuille V. Exploring the complementarity of fortification and dietary diversification to combat micronutrient deficiencies: a scoping review. Curr Developments Nutr. 2023;7(2):100033. 10.1016/j.cdnut.2023.100033. 10.1016/j.cdnut.2023.100033 PMC10111601 37180084

[5] Akinmoladun OF, Bamidele OP, Jideani VA, Nesamvuni CN. Severe acute malnutrition: the potential of non-peanut, non-milk ready-to-use therapeutic foods. Curr Nutr Rep. 2023;12(4):603–16. 10.1007/s13668-023-00505-9. 37897619 10.1007/s13668-023-00505-9 PMC10766793

[6] Rice AL, Sacco L, Hyder A, Black RE. Malnutrition as an underlying cause of childhood deaths associated with infectious diseases in developing countries. Bull World Health Organ. 2000;78:1207–21. 11100616 PMC2560622

[7] Vaitla B, Devereux S, Swan SH. Seasonal hunger: a neglected problem with proven solutions. PLoS Med. 2009;6(6):e1000101. 10.1371/journal.pmed.1000101. 19564900 10.1371/journal.pmed.1000101 PMC2696035

[8] WHO. Fact sheet: food safety. 2024 15 December 2025]; Available from: https://www.who.int/news-room/fact-sheets/detail/food-safety

[9] WHO. WHO estimates of the global burden of foodborne diseases. World Health Organization: Geneva; 2015.

[10] Alem AZ, Yeshaw Y, Liyew AM, Tessema ZT, Worku MG, Tesema GA, et al. Double burden of malnutrition and its associated factors among women in low and middle income countries: findings from 52 nationally representative data. BMC Public Health. 2023;23(1):1479. 10.1186/s12889-023-16045-4. 37537530 10.1186/s12889-023-16045-4 PMC10398981

[11] Nugent R, Levin C, Hale J, Hutchinson B. Economic effects of the double burden of malnutrition. Lancet. 2020;395(10218):156–64. 10.1016/S0140-6736(19)32473-0. 31852601 10.1016/S0140-6736(19)32473-0

[12] Botreau H, Cohen MJ. Gender inequality and food insecurity: a dozen years after the food price crisis, rural women still bear the brunt of poverty and hunger. Advances in Food Security and Sustainability. Elsevier; 2020. pp. 53–117. M.J. Cohen, Editor.

[13] Awuchi CG, Igwe VS, Amagwula IO. Ready-to-use therapeutic foods (RUTFs) for remedying malnutrition and preventable nutritional diseases. Int J Adv Acad Res. 2020;6(1):47–81.

[14] Osendarp S, Rogers B, Ryan K, Manary M, Akomo P, Bahwere P, et al. Ready-to-use foods for management of moderate acute malnutrition: considerations for scaling up production and use in programs. FoodNutr Bull. 2015;36(1):S59–64. 10.1177/15648265150361S110. 10.1177/15648265150361S110 25902616

[15] Das JK, Salam RA, Saeed M, Kazmi FA, Bhutta ZA. Effectiveness of interventions for managing acute malnutrition in children under five years of age in low-income and middle-income countries: a systematic review and meta-analysis. Nutrients. 2020;12(1):116. 10.3390/nu12010116. 31906272 10.3390/nu12010116 PMC7019612

[16] Ameye H, Glatzel K. Avoid ultra-processed foods, not food processing: why processed foods matter for nutrition security, economic growth, gender equality, and sustainability agendas in Africa. World Nutr. 2025;16(2):128–34. 10.26596/wn.2025162128-134.

[17] Lenters LM, Wazny K, Webb P, Ahmed T, Bhutta ZA. Treatment of severe and moderate acute malnutrition in low- and middle-income settings: a systematic review, meta-analysis and Delphi process. BMC Public Health. 2013;13(S3):S23. 10.1186/1471-2458-13-s3-s23. 24564235 10.1186/1471-2458-13-S3-S23 PMC3847503

[18] FAO/WHO. Report of the forty-first session of the codex committee on nutrition and foods for special dietary uses. Joint FAO/WHO Food Standards Programme: Rome; 2019.

[19] Borg B, Mihrshahi S, Laillou A, Sigh S, Sok D, Peters R, et al. Development and testing of locally-produced ready-to-use therapeutic and supplementary foods (RUTFs and RUSFs) in Cambodia: lessons learned. BMC Public Health. 2019;19(1):1200. 10.1186/s12889-019-7445-2. 31470824 10.1186/s12889-019-7445-2 PMC6717373

[20] Fetriyuna F, Purwestri RC, Jati I, Setiawan B, Huda S, Wirawan NN, et al. Ready-to-use therapeutic/supplementary foods from local food resources: Technology accessibility, program effectiveness, and sustainability, a review. Heliyon. 2023;9(12):e22478. 10.1016/j.heliyon.2023.e22478. 38046154 10.1016/j.heliyon.2023.e22478 PMC10686882

[21] Clark LF. Are innovative ready to use therapeutic foods more effective, accessible and cost-efficient than conventional formulations? A review. Outlook Agric. 2020;49(4):267–77. 10.1177/0030727020932184.

[22] Imdad A, Rogner JL, Francois M, Ahmed S, Smith A, Tsistinas OJ, et al. Increased vs. standard dose of iron in ready-to-use therapeutic foods for the treatment of severe acute malnutrition in a community setting: a systematic review and meta-analysis. Nutrients. 2022;14(15):3116. 10.3390/nu14153116. 35956294 10.3390/nu14153116 PMC9370784

[23] Moustiés C, Bourlieu-Lacanal C, Hemery YM, Baréa B, Villeneuve P, Servent A, et al. Nutritional quality of ready-to-use therapeutic foods: focus on lipid composition and vitamin content. OCL Oilseeds fats crops lipids. 2022;29. 10.1051/ocl/2022007.

[24] Borg B, Mihrshahi S, Griffin M, Sok D, Chhoun C, Laillou A, et al. Acceptability of locally-produced ready-to-use supplementary food (RUSF) for children under two years in Cambodia: a cluster randomised trial. Matern Child Nutr. 2019;15(3):e12780. 10.1111/mcn.12780. 30690869 10.1111/mcn.12780 PMC7198957

[25] Marchini M, Rosi A, Raia F, Bertolotti E, Scazzina F, Carini E. Acceptability of alternative ready-to-use therapeutic foods in acute malnutrition management-a systematic review. Int J Food Sci Nutr. 2022;73(8):993–1004. 10.1080/09637486.2022.2119213. 36064197 10.1080/09637486.2022.2119213

[26] Rachmadewi A, Soekarjo DD, Bait BR, Suryantan J, Noor R, Rah JH, et al. Ready-to-use therapeutic foods (RUTFs) based on local recipes are as efficacious and have a higher acceptability than a standard peanut-pased RUTF: a randomized controlled trial in Indonesia. Nutrients. 2023;15(14):3166. 10.3390/nu15143166. 37513584 10.3390/nu15143166 PMC10386054

[27] Thapa BR, Goyal P, Menon J, Sharma A. Acceptability and efficacy of locally produced ready-to-use therapeutic food nutreal in the management of severe acute malnutrition in comparison with defined food: a randomized control trial. FoodNutr Bull. 2017;38(1):18–26. 10.1177/0379572116689743. 10.1177/0379572116689743 28125907

[28] Aiello I, Kounnavong S, Vinathan H, Philavong K, Luangphaxay C, Soukhavong S, et al. Short-term acceptability of ready-to-use therapeutic foods in two provinces of Lao People’s Democratic Republic. Nutrients. 2023;15(17):3847. 10.3390/nu15173847. 37686879 10.3390/nu15173847 PMC10489829

[29] Rohwerder B. Behavioural economics/insights and health and nutrition in low- and middle-income countries. Institute of Development Studies: Brighton; 2017.

[30] Adhanom Ghebreyesus T. Using behavioural science for better health. Bull World Health Organ. 2021;99(11):755–755. 10.2471/blt.21.287387. 34737465 10.2471/BLT.21.287387 PMC8542276

[31] Dolan P, Hallsworth M, Halpern D, King D, Vlaev I. MINDSPACE: Influencing behaviour through public policy. 2010, Institute for Government: London.

[32] Michie S, van Stralen MM, West R. The behaviour change wheel: a new method for characterising and designing behaviour change interventions. Implement Sci. 2011;6(1):42. 10.1186/1748-5908-6-42. 21513547 10.1186/1748-5908-6-42 PMC3096582

[33] Vlaev I, Makki F. Applying behavioural insights in health: tackling key non communicable diseases. Warwick Business School: Coventry; 2018.

[34] Dellenbarger L, Schupp A, Liuzzo J, Andrews L. A sensory panel evaluation of selected species of finfish. J Food Prod Mark. 1993;1(3):37–44. 10.1300/J038v01n03_05.

[35] Makienko I, Schupp A, Prinyawiwatkul W, O’Neil CE, Pavon N. Consumers evaluate frozen and grilled ground beef and turkey product. J Food Prod Mark. 2005;11(1):23–33. 10.1300/J038v11n01_02.

[36] Misra SK, Fletcher SM, McWatters KH. Consumer acceptance of a new fast food. J Food Prod Mark. 1996;3(1):25–35. 10.1300/J038v03n01_03.

[37] Aldred Cheek K, Wansink B. Making it part of the package: edible packaging is more acceptable to young consumers when it is integrated with food. J Food Prod Mark. 2017;23(6):723–32. 10.1080/10454446.2017.1244793.

[38] Fetscherin M, Kiefer K, Braun-Herr ANS. Meatless revolution: exploring consumers’ perception of meat alternatives. J Food Prod Mark. 2024;30(5):154–69. 10.1080/10454446.2024.2377542.

[39] Rojas-Méndez JI, Ahmed SA, Claro-Riethmüller R, Spiller A. Acceptance of genetically modified foods with health benefits: a study in Germany. J Food Prod Mark. 2012;18(3):200–21. 10.1080/10454446.2012.666453.

[40] Lao Statistics Bureau. Lao Social Indicator Survey III-2023: key indicators report, January 2024. Lao Statistics Bureau: Vientiane; 2024.

[41] Lao Statistics Bureau and UNICEF. Lao social indicators survey III, 2023 (LSIS III, 2023): survey findings report. Lao Statistics Bureau and United Nations Children’s Fund: Vientiane; 2025.

[42] Keokenchanh S, Kounnavong S, Tokinobu A, Midorikawa K, Ikeda W, Morita A, et al. Prevalence of anemia and its associate factors among women of reproductive age in Lao PDR: evidence from a nationally representative survey. Anemia. 2021;2021:1–9. 10.1155/2021/8823030. 10.1155/2021/8823030 PMC7822650 33520310

[43] Phuong H, Thuy Nga T, Mathisen R, Nguyen M, Thi Hop L, Bao Hoa DT, et al. Development and implementation of a locally produced ready-to-use therapeutic food (RUTF) in Vietnam. FoodNutr Bull. 2014;35(2):S52–6. 10.1177/15648265140352S108. 10.1177/15648265140352S108 25069294

[44] Kibe M, Mizuno Y, Masuoka H, Kosaka S, Natsuhara K, Hirayama K, et al. Transition to a market economy and chronic psychosocial stress in northern Laos: An exploratory study of urinary free cortisol in rural residents. Am J Hum Biology. 2023;e23976. 10.1002/ajhb.23976. 10.1002/ajhb.23976 37577830

[45] World Bank. World databank. 2025 15 December 2025]; Available from: https://databank.worldbank.org/home.aspx

[46] Rizaldo QV, Inphonephong S, Phounvisouk L, Dubois M. Diet diversity among women in Attapeu Province, Lao PDR. International Water Management Institute: Colombo; 2024.

[47] Kounnavong T, Sato M, Turner C, Ferguson E, Xayavong H, Vonglokham M, et al. Drivers of food acquisition practices among adolescents in suburban food environments of Lao People’s Democratic Republic. Global Health Action. 2025;18(1):2451475. 10.1080/16549716.2025.2451475. 39898692 10.1080/16549716.2025.2451475 PMC11792158

[48] Haenssgen MJ, Charoenboon N, Xayavong T, Lathsamee T, Leepreecha P, Deharo E, et al. Conceptualising food environments as social activity spaces: Insights from lived experience research in Thailand and Laos. Volume 96. Health & Place; 2025. p. 103578. 10.1016/j.healthplace.2025.103578. 10.1016/j.healthplace.2025.103578 41205440

[49] Coulombe H, Epprecht M, Pimhidzai O, Sisoulath V. Where are the poor? Lao PDR 2015 census-based poverty map: province and district level results. Ministry of Planning and Investment: Vientiane; 2016.

[50] Sigh S, Roos N, Sok D, Borg B, Chamnan C, Laillou A, et al. Development and acceptability of locally made fish-based, ready-to-use products for the prevention and treatment of malnutrition in Cambodia. FoodNutr Bull. 2018;39(3):420–34. 10.1177/0379572118788266. 10.1177/0379572118788266 30092653

[51] Goyal A, Tanwar B, Kumar Sihag M, Sharma V. Sacha inchi (Plukenetia volubilis L.): an emerging source of nutrients, omega-3 fatty acid and phytochemicals. Food Chem. 2022;373:131459. 10.1016/j.foodchem.2021.131459. 34731811 10.1016/j.foodchem.2021.131459

[52] Kodahl N. Sacha inchi (Plukenetia volubilis L.)—from lost crop of the Incas to part of the solution to global challenges? Planta. 2020;251(4):80. 10.1007/s00425-020-03377-3. 32185506 10.1007/s00425-020-03377-3

[53] Wang S, Zhu F, Kakuda Y. Sacha inchi (Plukenetia volubilis L.): nutritional composition, biological activity, and uses. Food Chem. 2018;265:316–28. 10.1016/j.foodchem.2018.05.055. 29884388 10.1016/j.foodchem.2018.05.055

[54] Radio Free Asia. Lao government increases minimum wage for third time since 2018. 2023 5 February 2024]; Available from: https://www.rfa.org/english/news/laos/minimum-wage-10022023135555.html

[55] Sidell DR, Kim IA, Coker TR, Moreno C, Shapiro NL. Food choking hazards in children. Int J Pediatr Otorhinolaryngol. 2013;77(12):1940–6. 10.1016/j.ijporl.2013.09.005. 24113156 10.1016/j.ijporl.2013.09.005

[56] Gracia A. Consumers’ preferences for a local food product: a real choice experiment. Empirical Economics. 2014;47(1):111–28. 10.1007/s00181-013-0738-x.

[57] Motoki K, Saito T, Onuma T. Eye-tracking research on sensory and consumer science: A review, pitfalls and future directions. Food Res Int. 2021;145:110389. 10.1016/j.foodres.2021.110389. 34112392 10.1016/j.foodres.2021.110389

[58] Husić-Mehmedović M, Omeragić I, Batagelj Z, Kolar T. Seeing is not necessarily liking: advancing research on package design with eye-tracking. J Bus Res. 2017;80:145–54. 10.1016/j.jbusres.2017.04.019.

