# Supplementary material for: Consumer acceptance of a locally produced nutritive food bar in Lao PDR
Source: BMC Nutr. 2026 Jul 3;12:171. doi: 10.1186/s40795-026-01385-2 (PMC13491913; doi:10.1186/s40795-026-01385-2)
Supplement: Supplementary file 1 [file 40795_2026_1385_MOESM1_ESM.pdf]

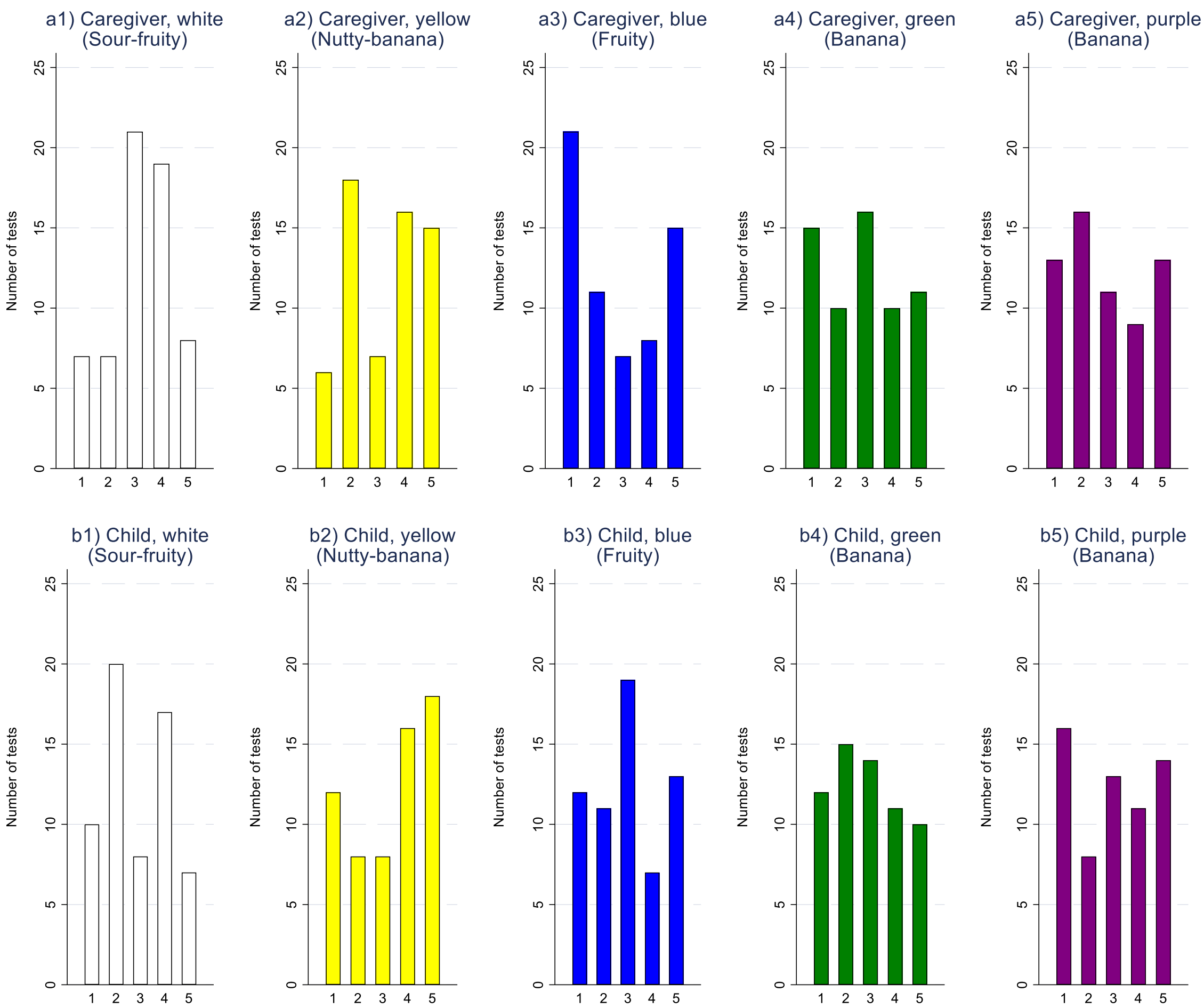

Fig. S1. Bar sort order across organoleptic tests with adult caregivers and children.

Notes. N = 124 (62 adults, 62 children). Colours corresponding to food bar codes.

Nutrilao questionnaire

**1**	**Household Identification**	
1.1	Village	Ban Nayang ....5<br>Ban Na-ngeun ....6<br>Ban Boong-phao ....7<br>Ban Hadxai 8
1.2	Household number	Number: ______
1.3	Enumerator name	Enumerator name: __________
1.4	Household coordinates — a) Latitude	[coordinates entered automatically]
	Household coordinates — b) Longitude	[coordinates entered automatically]
	**Statement of consent (Respondent will receive participant information sheet and verbal consent will be taken)**<br>Thank you for participating. You will receive a small token of gratitude for your participation at the end of the interview.	
1.5	Date of interview	[date entered automatically]
1.6	Time of interview	[time entered automatically]

**2**	**Caregiver testing**<br>Enumerator: *We will start by testing the food bars with you as the caregiver of your child. We have five bars, and we will get your opinion on the one-by-one. I am happy to eat them with you if you would like some reassurance that they are safe to eat.*	
2.1	What is your name?	Respondent name: _________________________
2.2	How old are you? [in years]	Age in years: ______
2.3	What is your gender?	Non-binary / diverse ....2<br>Female ....1<br>Male 0
2.4	What is the highest grade of schooling that you completed?	No formal education ....0<br>Primary / elementary ....1<br>Lower secondary ....2<br>Upper secondary ....3<br>Post-secondary / vocational ....4<br>Tertiary / university ....5<br>Other (specify: _________) ....8<br>Don't know / prefer not to answer 9
	[Randomise bars, use colour stickers to distinguish them]<br>[Present bars on a plate]<br>[Give participant water to drink between each bar]	1: [blue] Classic<br>2: [green] Classic-energy<br>3: [yellow] Classic-mung<br>4: [purple] Classic-peanut<br>5: [white] Mung-hibiscus
2.5	Could you please tell me how good or how bad you would rate the bar you just have been eaten according to…	[separately for each bar 1-5]
2.5.1	Colour	Very bad 1 · Bad 2 · So-so 3 · Good 4 · Very good 5<br>No opinion / prefer not to answer 9
2.5.2	Smell	Very bad 1 · Bad 2 · So-so 3 · Good 4 · Very good 5<br>No opinion / prefer not to answer 9
2.5.3	Taste	Very bad 1 · Bad 2 · So-so 3 · Good 4 · Very good 5<br>No opinion / prefer not to answer 9
2.5.4	Appearance (looks delicious)	Very bad 1 · Bad 2 · So-so 3 · Good 4 · Very good 5<br>No opinion / prefer not to answer 9

2.5.5 Chewiness	Very bad 1 | Bad 2 | So-so 3 | Good 4 | Very good 5<br>No opinion / prefer not to answer 9
2.5.6 Hardness	Very bad 1 | Bad 2 | So-so 3 | Good 4 | Very good 5<br>No opinion / prefer not to answer 9
2.5.7 Overall	Very bad 1 | Bad 2 | So-so 3 | Good 4 | Very good 5<br>No opinion / prefer not to answer 9
**2.6** Now I would like to ask you for your overall judgement across these five food bars.	
2.6.1 Which bar did you like most?	[blue] Classic...1<br>[green] Classic-energy..2<br>[yellow] Classic-mung ....3<br>[purple] Classic-peanut...4<br>[white] Mung-hibiscus 5
2.6.2 Which bar did you like least?	[blue] Classic...1<br>[green] Classic-energy..2<br>[yellow] Classic-mung ....3<br>[purple] Classic-peanut...4<br>[white] Mung-hibiscus 5

3 Child testing

Enumerator: *Thank you. We will now let your child try and rate the different bars. The process and questions are exactly the same as before.*

**3.1** What is the child's name?	Child name: ______________________________
**3.2** How old is the child? [in years]	Age in years: _______
**3.3** What is the child's gender?	Non-binary / diverse ....2<br>Female ....1<br>Male 0
**3.4** Is the child currently going to school?	Yes...1<br>No..0<br>Don't know / prefer not to answer 9
[Randomise bars, use colour stickers to distinguish them]<br>[Present bars on a plate]<br>[Give participant water to drink between each bar]	1: [blue] Classic<br>2: [green] Classic-energy<br>3: [yellow] Classic-mung<br>4: [purple] Classic-peanut<br>5: [white] Mung-hibiscus
**3.5** Could you please tell me how good or how bad you would rate the bar you just have been eaten according to…	[separately for each bar 1-5]
3.5.1 Colour	Very bad 1 | Bad 2 | So-so 3 | Good 4 | Very good 5<br>No opinion / prefer not to answer 9
3.5.2 Smell	Very bad 1 | Bad 2 | So-so 3 | Good 4 | Very good 5<br>No opinion / prefer not to answer 9
3.5.3 Taste	Very bad 1 | Bad 2 | So-so 3 | Good 4 | Very good 5<br>No opinion / prefer not to answer 9
3.5.4 Appearance (looks delicious)	Very bad 1 | Bad 2 | So-so 3 | Good 4 | Very good 5<br>No opinion / prefer not to answer 9

3.5.5	Chewiness	Very bad 1 · Bad 2 · So-so 3 · Good 4 · Very good 5<br>No opinion / prefer not to answer 9
3.5.6	Hardness	Very bad 1 · Bad 2 · So-so 3 · Good 4 · Very good 5<br>No opinion / prefer not to answer 9
3.5.7	Overall	Very bad 1 · Bad 2 · So-so 3 · Good 4 · Very good 5<br>No opinion / prefer not to answer 9
**3.6**	Now I would like to ask you for your overall judgement across these five food bars.	
3.6.1	Which bar did you like most?	[blue] Classic...1<br>[green] Classic-energy..2<br>[yellow] Classic-mung ....3<br>[purple] Classic-peanut ....4<br>[white] Mung-hibiscus 5
3.6.2	Which bar did you like least?	[blue] Classic...1<br>[green] Classic-energy..2<br>[yellow] Classic-mung ....3<br>[purple] Classic-peanut ....4<br>[white] Mung-hibiscus 5

4 Willingness to use ready-to-use-foods

Enumerator: *Thank you, in the rest of the interview, I will just ask you a few more questions about the food you consume. There are no right or wrong answers, we really just want to hear what you think so we can learn from you.*
For now, let us focus on "ready-to-use foods," which are foods that do not need any preparation time, or very little preparation time. We will focus here on those that you can buy in a shop and that come in a package – and can be eaten directly from the package, for example instant noodle soup, cookies, carbonated drinks, or sweets. We are not considering fruits and vegetables for now.

4.1	In your household, does any family member at least sometimes consume ready-to-use foods and drinks?	Yes...1<br>No...0<br>Don't know / prefer not to answer 9
4.2	[If yes to 4.1] Can you name all the household members who consume ready-to-use foods and drinks?	Household member 1: ________________<br>Household member 2: ________________<br>Household member n: ________________
4.2.1	[for each person listed in 4.2] How old is [household member n]?	Age in years: _______
4.2.2	[for each person listed in 4.2] What is the gender of [household member n]?	Non-binary / diverse ....2<br>Female ....1<br>Male 0
4.2.3	[for each person listed in 4.2] How often does [household member n] consume any ready-to-use foods and drinks?	Daily or more often...5<br>Few times per week...4<br>Few times per month...3<br>Less than monthly...2<br>Never / almost never ....1<br>Don't Know / Prefer not to answer 9
**4.3**	Now let us talk about your entire household. Which of the following ready-to-use foods and drinks are being used by any of your household family members? [consider the family as a whole, regardless of whether people eat/drink these foods individually or together]	
4.3.1	Carbonated drinks	Daily or more often...5<br>Few times per week...4<br>Few times per month...3<br>Less than monthly...2<br>Never / almost never ....1<br>Don't Know / Prefer not to answer 9
4.3.2	Instant noodles that you prepare yourself (e.g. at home)	Daily or more often...5<br>Few times per week...4<br>Few times per month...3<br>Less than monthly...2<br>Never / almost never ....1<br>Don't Know / Prefer not to answer 9
4.3.3	Sweets	Daily or more often...5<br>Few times per week...4<br>Few times per month...3<br>Less than monthly...2<br>Never / almost never ....1<br>Don't Know / Prefer not to answer 9

4.3.4	Cookies / cakes	Daily or more often ....5<br>Few times per week ....4<br>Few times per month ....3<br>Less than monthly ....2<br>Never / almost never ....1<br>Don't Know / Prefer not to answer 9
4.3.5	Salty snacks	Daily or more often ....5<br>Few times per week ....4<br>Few times per month ....3<br>Less than monthly ....2<br>Never / almost never ....1<br>Don't Know / Prefer not to answer 9
4.3.6	Are there other ready-to-use foods and drinks that you are thinking about? If so, please specify what they are and how often your household consumes them: ____________________	Daily or more often ....5<br>Few times per week ....4<br>Few times per month ....3<br>Less than monthly ....2<br>Never / almost never ....1<br>Don't Know / Prefer not to answer 9
4.4	For you personally, I would now like to know which factors make you decide for or against ready-to-use foods – even if you never ate or drank them, I would still like to hear your experiences and opinions. What role do the following factors play in deciding for or against ready-to-use foods?	
4.4.1	Convenience	✘ Reason against ready-to-use food/drink 1 · ● Neutral / no influence 2 · ✓ Reason for ready-to-use food/drink 3<br>No opinion / prefer not to answer 9
4.4.2	To quench thirst or hunger	✘ Reason against ready-to-use food/drink 1 · ● Neutral / no influence 2 · ✓ Reason for ready-to-use food/drink 3<br>No opinion / prefer not to answer 9
4.4.3	Availability in my village	✘ Reason against ready-to-use food/drink 1 · ● Neutral / no influence 2 · ✓ Reason for ready-to-use food/drink 3<br>No opinion / prefer not to answer 9
4.4.4	Taste	✘ Reason against ready-to-use food/drink 1 · ● Neutral / no influence 2 · ✓ Reason for ready-to-use food/drink 3<br>No opinion / prefer not to answer 9
4.4.5	Price	✘ Reason against ready-to-use food/drink 1 · ● Neutral / no influence 2 · ✓ Reason for ready-to-use food/drink 3<br>No opinion / prefer not to answer 9
4.4.6	Healthiness	✘ Reason against ready-to-use food/drink 1 · ● Neutral / no influence 2 · ✓ Reason for ready-to-use food/drink 3<br>No opinion / prefer not to answer 9
4.4.7	When spending time with friends	✘ Reason against ready-to-use food/drink 1 · ● Neutral / no influence 2 · ✓ Reason for ready-to-use food/drink 3<br>No opinion / prefer not to answer 9
4.5	Approximately how many Kip does your household spend on ready-to-use foods per week?	LAK: ___________ per week
4.6	Thinking of the food bar that you just tasted and liked the most: How much would you be willing to pay for one such bar?	LAK: ___________ per bar

**4.7** If this food bar was available at a shop in the village, would you buy it?	Yes....1<br>No....0<br>Don't know / prefer not to answer....9
**4.8** If this food bar was available at school, would you let your child buy it?	Yes....1<br>No....0<br>Don't know / prefer not to answer....9
**5 Nutrition knowledge, attitudes, and behaviors**<br>Enumerator: *Thank you, we are now in the final, short part of our interview.*	
**5.1** Have you ever heard about vitamins and minerals? [**if no, skip to** 5.2]	Yes....1<br>No....0<br>Don't know / prefer not to answer....9
**5.1.1** Could you name any specific vitamins or minerals?	Specify: ________________________
**5.1.2** Could you try to explain what vitamins and minerals do to the human body?	Specify: ________________________
**5.1.3** Do you think that vitamins and minerals are part of your normal food?	Yes....1<br>No....0<br>Don't know / prefer not to answer....9
**5.1.4** Do you think that all foods contain the same vitamins and minerals?	Yes....1<br>No....0<br>Don't know / prefer not to answer....9
**5.1.5** Do you think that vitamins and minerals are important for humans?	Yes....1<br>No....0<br>Don't know / prefer not to answer....9
**5.1.5.1** [if yes to 5.1.5] Can you please specify how important?	Not important....0<br>A little bit important....1<br>Important....2<br>Very important....3<br>Don't know / prefer not to answer....9
[if yes to 5.1.5] Based on your knowledge and experiences, do you believe that vitamins are minerals are important for:	
**5.1.5.2** Learning or becoming smart	Yes....1<br>No....0<br>Don't know / prefer not to answer....9
**5.1.5.3** Good taste of food	Yes....1<br>No....0<br>Don't know / prefer not to answer....9
**5.1.5.4** Growth of children	Yes....1<br>No....0<br>Don't know / prefer not to answer....9
**5.1.5.5** Energy and strength for work	Yes....1<br>No....0<br>Don't know / prefer not to answer....9
**5.1.5.6** A desirable skin colour	Yes....1<br>No....0<br>Don't know / prefer not to answer....9
**5.1.5.7** Healthy babies	Yes....1<br>No....0<br>Don't know / prefer not to answer....9
**5.1.5.8** Getting well after sickness	Yes....1<br>No....0<br>Don't know / prefer not to answer....9
**5.1.6** What do you think is the best way for people to get vitamins and minerals?	Specify: ________________________
**5.2** Have you taken any supplements before? (probe: as children, teenagers, for pregnancy)	Yes....1<br>No....0<br>Don't know / prefer not to answer....9
**5.3** Have you ever heard about food fortification?	Yes....1<br>No....0<br>Don't know / prefer not to answer....9
**5.3.1** [If yes] Can you name a food that is fortified with vitamins or minerals?	Specify: ________________________

5.4 Have you ever received or seen nutrition information for the child? If so, from which sources? [*mark all that apply*]	No ....0<br><br>Family members ສະມາຊິກໃນຄອບຄົວ ....1<br>Other villagers ສະມາຊິກໃນກຸ່ມບ້ານ....................2<br>Village leader / loudspeaker ຜູ້ນຳຂັ້ນບ້ານ/ການປະກາດໂຫລະໂຄ່ງ ....3<br>Temple ວັດ....................4<br>Church ໂບດ....................5<br>Lao Front ແນວລາວສ້າງຊາດ ....6<br>Lao Women's Union ສະຫະພັນແມ່ຍິງ....................7<br>Lao Youth Union ຊາວໜຸ່ມ....................8<br>Other authorities ແລະ ອຳນາດການປົກຄອງ ອື່ນໆ....................9<br><br>Posters ໃບປິວ ....10<br>Newspaper ໜັງສືພິມ ....11<br>TV / radio ໂທລະພາບ/ວິທະຍຸ....................12<br>Social media ສື່ສັງຄົມອອນລາຍ ....13<br>Other online websites ເວັບໄຊອອນລາຍອື່ນໆ....................14<br><br>Shop / vendor selling drugs ຮ້ານຄ້າ/ຮ້ານຂາຍຢາທົ່ວໄປ ....20<br>Traditional healer ໝໍພື້ນເມືອງ/ໝໍຜີ ....21<br>Pharmacist ແພດການຢາ ....22<br>Private clinic/hospital ຄລີນິກເອກະຊົນ/ໂຮງໝໍ....................23<br>NGO clinic/service ຄລີນິກຂອງອົງການຈັດຕັ້ງສາກົນ/ການບໍລິການ ......24<br>Health centre ສຸກສາລາ/ໂຮງໝໍນ້ອຍ ....25<br>Public hospital ໂຮງໝໍໃຫຍ່ (ໂຮງໝໍລັດ)....................26<br>Village health volunteer ອາສາສະໝັກສາທາລະນະສຸກບ້ານ (ອສບ).......27<br>Unregistered/local/informal/retired doctor....................28<br>ໝໍບໍ່ມີໃບຢັ້ງຢືນແພດ/ທ້ອງຖິ່ນ/ບໍ່ເປັນທາງການ/ໝໍບຳນານ<br>Traditional birth assistant ໝໍຕຳແຍ ....29<br><br>Others 99
5.5 Have you or someone you know ever provided micronutrient powder, paste, bars, or milk for your child, for example Superkid or F100 therapeutic milk?<br>[*mark all that apply*]	Yes: micronutrient powder....................1<br>Yes: micronutrient paste ....2<br>Yes: micronutrient bar ....3<br>Yes: micronutrient milk....................4<br>Yes: Other (specify: __________) ....8<br><br>No....................0<br>Don't know / prefer not to answer 9
**6 Questionnaire End**	
Thank you very much for participating in this survey.	
6.1 Were there any unusual circumstances during the interview?	Please describe: ______________________________
